# The Efficacy of Night Bracing in the Treatment of Adolescent İdiopathic Scoliosis: A Systematic Review

**DOI:** 10.3390/jcm13133661

**Published:** 2024-06-23

**Authors:** Tuğba Kuru Çolak, Elif Elçin Dereli, Burçin Akçay, Adnan Apti, Santiago Lasa Maeso

**Affiliations:** 1 Department of Physiotherapy and Rehabilitation, Faculty of Health Sciences, Marmara University, Istanbul 34854, Turkey; 2 Department of Physiotherapy and Rehabilitation, Faculty of Health Sciences, Istanbul Bilgi University, Istanbul 34060, Turkey; 3 Department of Physiotherapy and Rehabilitation, Faculty of Health Sciences, Bandırma Onyedi Eylül University, Balıkesir 10250, Turkey; 4 Department of Physiotherapy and Rehabilitation, Faculty of Health Sciences, Istanbul Kültür University, Istanbul 34158, Turkey; a.apti@iku.edu.tr; 5 Clinica De La Escoliosis, Montevideo 11000, Uruguay; santiagolasa@gmail.com

**Keywords:** adolescent, brace, scoliosis, spine

## Abstract

**Background/Objectives:** The effectiveness of night braces alone or in combination with other treatments for adolescent idiopathic scoliosis remains unclear. This systematic review study aimed to review and analyze the available literature to determine whether night braces are an effective treatment for idiopathic scoliosis. **Methods:** A total of 162 databases, including Cochrane Library (reviews, protocols, trials), Web of Science, PubMed, Medline, Scopus, PEDro, CINAHL (EBSCO), Ovid and Google Scholar, were searched for published articles from inception to February 2024. The available literature was screened by the following terms: “scoliosis and night-time brace”, “scoliosis and night brace”, “scoliosis and part-time bracing”, “scoliosis and Providence” and “scoliosis and Charleston”. **Results:** Twenty studies were included; only one study was a randomized controlled trial, and most of the studies were retrospectively designed. Providence, Charleston and Boston braces were used as night braces. The Cobb angle was evaluated in all studies, and Cobb angle change after treatment and surgical treatment rates were the parameters that were evaluated the most. In one study, the angle of trunk rotation, quality of life, perception of spinal appearance, and physical activity level were measured. In one study, sagittal plane assessments were performed in addition to the Cobb angle. **Conclusions:** The results of this review suggest that there is no evidence to support the use of night braces in the treatment of adolescent idiopathic scoliosis. Randomized controlled trials with a well-designed methodology are needed to determine the efficacy of night braces.

## 1. Introduction

High-quality data support the use of rigid full-time bracing in patients diagnosed with adolescent idiopathic scoliosis (AIS) prior to full skeletal maturation [1,2]. In the study of Weinstein et al. evaluating the effects of different thoracolumbar braces in the treatment of AIS, it was shown that brace treatment was effective with a success rate of 72% in patients who received brace treatment compared to patients who received only observation [2]. In the Gensingen brace, a CAD-based asymmetric brace, the success rate has been reported between 86 to 96% for curvatures between 25 and 40° as well as for curvatures exceeding 40° [3,4]. Also, a recent systematic review conducted by Babaee et al. provides evidence for the effectiveness of bracing in controlling the progression of AIS in curves greater than 40° [5].

Today, the use of a brace can be recommended to the patient as a full-time, part-time, or only at night treatment. Wearing a brace for 20 h or more is considered full time, while wearing one for 12 to 20 h is considered part time. Typically, night-time braces are worn for eight hours at a time [1,6]. For the effectiveness of brace treatment, full-time brace use and patient compliance are important, especially in periods of rapid growth and progression [1,7,8,9,10,11]. However, some authors nowadays suggest that using the brace only at night produces similar effects compared to full-time use [12,13].

The effectiveness of night braces has been assessed in some systematic reviews, and in some of them, it has been concluded that they are effective [14,15]. In contrast, Ruffilli et al. analyzed seven studies until 2020 and concluded that “The current available literature does not permit us to draw conclusions about night-time braces” [16]. Moreover, the study by Bretschneider et al. suggested that daytime or full-time Chêneau brace use was more effective in reducing curvature than the Charleston brace used only at night [17].

According to these contradicting suggestions, high-quality research is needed to comprehensively evaluate the efficacy and potential advantages and disadvantages of night bracing in the treatment of AIS. In particular, it should be taken into consideration that the main aim of bracing is to interfere with the asymmetrical loading by gravity during daytime activities and that the curve may naturally decrease at night due to the relaxation of muscles and ligaments while resting in horizontal positions.

Overall, the efficacy of night bracing in the treatment of adolescent idiopathic scoliosis remains a topic of ongoing research and debate. However, academically and clinically, it was observed that most of the studies included in these systematic reviews [14,15] had a retrospective design and were characterized by methodological deficits. This systematic review was designed with the hypothesis that night braces may be effective in the treatment of AIS when used alone or in combination with other treatments. The aim of this study was to reveal the efficacy of night braces in the treatment of adolescent idiopathic scoliosis by actually examining the content of existing studies, to conduct a systematic review, and to shed light on future studies.

## 2. Materials and Methods

### 2.1. Search Strategy

The search strategy was standardized using the Preferred Reporting Items for Systematic Reviews and Meta-Analyses (PRISMA) checklist. Relevant published articles on the use of night braces for treating scoliosis and idiopathic scoliosis in adolescents were examined by three independent researchers. In this context, the Online Library System analyzed the electronic databases of Istanbul Bilgi, Marmara and Bandirma Onyedi Eylül Universities. These libraries have a total of 162 databases, including Cochrane Library (reviews, protocols, trials), Web of Science, PubMed, Medline, Scopus, PEDro, CINAHL (EBSCO), Ovid and Google Scholar. Regarding search terms, databases were searched for published articles from inception to April 2024. By reading the abstracts and titles, researchers independently filtered the search results. Initially, duplicate results were examined and excluded. Their inclusion was independently determined after obtaining the full texts of the possibly pertinent studies. The articles were discussed among the researchers in the event that there were disagreements among the authors.

### 2.2. Selection of the Studies

The available literature was screened using the terms “scoliosis and night-time brace,” “scoliosis and night brace,” “scoliosis and part-time bracing,” “scoliosis and Providence,” and “scoliosis and Charleston.”

Only publications in English, Turkish and Spanish were considered. Studies that sampled participants with idiopathic scoliosis and diagnosed them with radiographic assessment included interventions, including night-time bracing and presenting objective treatment results such as Cobb angle, angle of rotation, or other outcome measurements. Studies that sampled participants with <10° Cobb angle, studies included patients who used different braces during the day and night at the same time, studies that included only cases in which certain improvements were achieved in the brace, studies including soft braces, retracted studies, case reports, study protocols, conference abstracts and thesis studies were excluded.

The first and second reviewers independently evaluated the study designs and evidence levels in accordance with the Center of Evidence-Based Medicine in Oxford guidelines for therapeutic studies [18]. Level I was assigned to systematic reviews of randomized controlled trials (RCTs) and RCTs with narrow confidence intervals; Level II was assigned to prospective controlled studies and RCTs of lower quality; Level III was assigned to retrospective controlled studies; and Level IV was assigned to uncontrolled studies.

## 3. Results

### 3.1. Descriptive Data

**Figure 1:**
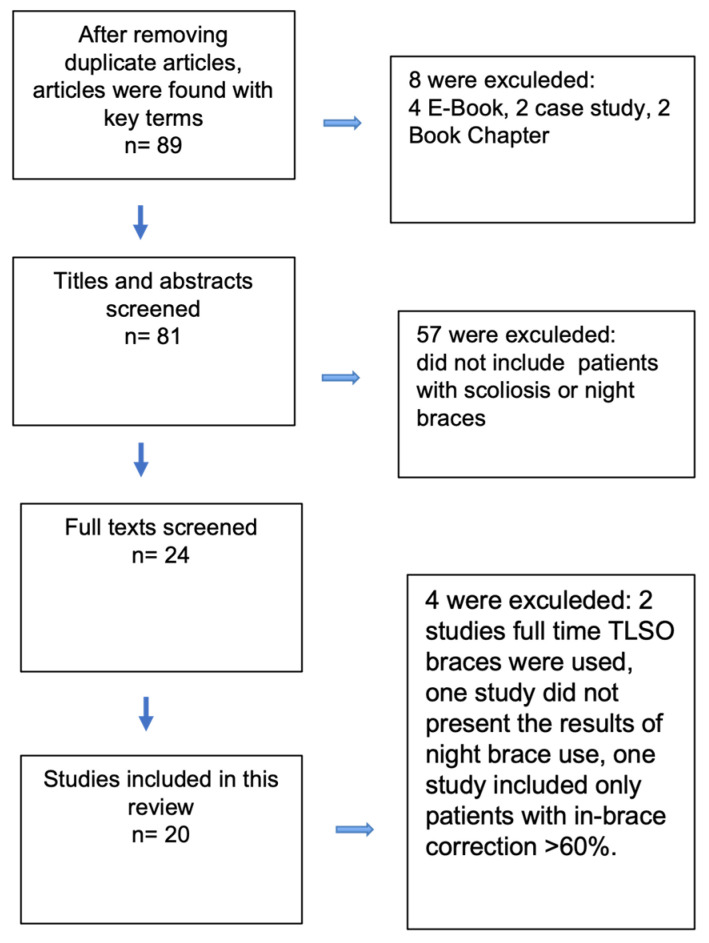
Flow chart of study evaluation and selection process.

After removing duplicate articles, a total of 89 articles were found between 1960 and February 2024. After abstract and full-text reviews, 20 studies (21 published articles) were included in our study (Figure 1) [13,19,20,21,22,23,24,25,26,27,28,29,30,31,32,33,34,35,36,37,38]. The two included articles were from the same randomized controlled trial. One included 6-month results from the same trial [33], and the other included the trial’s full results [38].

The study characteristics are summarized in Table 1. The 20 studies that meet the inclusion criteria were published between 1997 and 2024 and were conducted in eight countries. Five studies are from Denmark [29,32,34,36,37], and five studies are from the USA [19,22,23,28,31] (Table 1). Most of the studies (14 studies) are retrospective studies with level IV evidence. Only one study from Sweden [33,38] is a randomized controlled study with level Ib evidence. In one study, the study design is not specified [12], and the others are uncontrolled prospective studies [13,20,23,25].

A total of 2764 participants were included in the studies (Table 1). All patients included in the studies were diagnosed with idiopathic scoliosis. Overall, 1897 of these patients used a Providence or Charleston night brace. In a randomized controlled trial, 45 patients used a Boston brace at night [33,38]. In one study, a Boston brace was used in 168 patients, but it was reported that patients wore the brace for 12 to 13 h a day, but it is not clear at what time of day the brace was worn [30]. Most of the articles stated the duration of wearing the brace as 8 h per day. However, this time of use could have been objectively measured.

In several studies, the Boston brace [19,22,25,32,35] or TLSO brace [21,24,26] was used in studies with a control group, and the Milwaukee brace [21,22] was used as a third group in two studies that used the TLSO and Boston brace in the control group. In the only randomized controlled study, scoliosis-specific exercises and physical activity recommendations were applied in the control groups [33,38].

Two studies included 1850 females (66.9%), and gender characteristics were not presented in them [21,30]. Only male patients were included in the study by Karol et al. [22].

Although other studies reported the inclusion of patients diagnosed with adolescent idiopathic scoliosis (>10 years), one study [13] included patients aged between 4 and 15. Furthermore, the only available randomized controlled trial included patients aged 9 to 17 [33,38].

Studies generally included patients with a Risser value of 0–2. However, four studies included patients with Risser values between 0 and 3 [13,22,25,35]. Two studies, that by Hegard et al. [34,37] and a randomized controlled study [34,38], included patients with Risser values between 0 and 4. The lowest Cobb angle was 18 degrees, and the highest Cobb angle was 55 degrees in the patients included in the studies.

### 3.2. Outcome Measurements

When the studies’ outcome measurements were reviewed, it was determined that all studies involved measuring the Cobb angle and the amount of change in Cobb angle; in other words, the curve progression rate was evaluated. In the studies, progression was defined as an increase in radiographic Cobb angle >5°, while a decrease in Cobb angle of more than 5° was defined as success.

In most of the studies, the rate of surgical treatment was also evaluated. Only Karol et al. and Capek et al. evaluated brace compliance [22,35]. Vicente et al. presented the results of POTSI (Posterior Trunk Symmetry Index) and deformity index evaluation at the beginning of their study but did not specify how these values changed after treatment [13]. This systematic review’s only randomized controlled trial included assessments of Cobb angle, ATR (angle of trunk rotation), quality of life, perception of spinal appearance, and physical activity level [33]. However, in the other article in which the study results were published, these evaluations were not reported, and only the curve progression rate was presented [33,38]. A retrospective analysis of 146 patients by Heegaard et al. included sagittal plane assessment as well as Cobb angle change [36]. 

## 4. Discussion

The effectiveness of night braces alone or combined with other treatments for adolescent idiopathic scoliosis remains unclear. This systematic review study aimed to review and analyze the available literature to determine whether night braces are an effective treatment for idiopathic scoliosis at risk for being progressive. A total of twenty original research studies were included and analyzed. There was only one randomized controlled study, which two published articles were in reference to [33,38]. 

In patients with curves greater than 20° ± 5° Cobb, who are still growing (Risser 0 to 3), and who have deformity progression or an increased risk of worsening, bracing is advised unless a clinician with expertise in conservative treatment of spinal deformities determines otherwise [6,7]. All studies reported changes in Cobb angles, as a measure of deformity. Patients with Cobb angles ranging from 25 to 40 degrees of curvature were included in ten of the studies. Among the patients included in the studies, the lowest Cobb angle was 18 degrees, and the highest Cobb angle was 55 degrees. 

Although the mean baseline Cobb angle values differed in the studies, a 48–81.2% success rate was reported. Katz et al. and Howard et al. reported that Boston and TLSO braces worn full time were more effective than night braces [19,21]. Karol et al. and Gepstein et al. reported that night braces had similar effects to other brace types [22,24]. Howard et al. compared the effects of TLSO, Charleston and Milwakee braces and concluded that “The thoracolumbosacral orthosis was superior at preventing curve progression in adolescent idiopathic scoliosis.” [21]. Yjonen and Janicki emphasized that night braces may be effective in small curves [25,26]. The only RCT that could be included in this systematic review reported that scoliosis-specific exercises, physical activity and night bracing had similar effects on Cobb angle improvement during the first six months of treatment [33]. In the end-result article of the same study but with a different first author, the success of the night brace was reported as 76% [38]. The article presenting the result did not present the results of the parameters evaluated, unlike the article presenting the results of the first six months [38]. The most important methodological problem in this randomized controlled trial is that the exercises or physical activity recommended to the patients must be controlled. The authors also did not present the Cobb angle values at the beginning and end of the treatment [33,38]. 

A study reporting that night braces are highly effective in the literature could not be included in this review [12]. Only patients with an initial in-brace Cobb angle correction greater than 60% were included by the authors [8]. Unsurprisingly, the results outperform those of other published cohorts when patients with the best possible in-brace correction are the only ones included and when X-rays are taken in the supine position [12]. The exclusive inclusion of patients with a correction effect of > 60% (X-rayed in horizontal position) disqualifies this study for any comparative analysis (selection bias). The paper by Simony et al. needs to include information regarding the results of patients whose correction effect was less than 60 degrees. Since the Risser sign has not been documented, the authors cannot confirm that each patient satisfies the SRS inclusion requirements (Risser 0–2) [12]. The different patient cohorts presented in this study had an average age exceeding 13 years, so we may assume the average patient from this study was at a Risser 2–3, which would represent a more mature sample. Within the BRAIST study by Weinstein and colleagues [2], as well as in other studies following the inclusion criteria for studies on bracing, the average age is about 12.6 years [29,39]. These facts, among others, were outlined in a letter to the editor from Potts [40]. 

Idiopathic scoliosis is called adolescent idiopathic scoliosis if a patient is diagnosed at the age of 10 years or older [6,7]. In the criteria recommended by the Scoliosis Research Society (SRS) to investigate the effectiveness of brace treatment, it was reported that the age of the patients should be older than 10 years [41]. In the majority of studies, patients with idiopathic scoliosis over the age of 10 years were included. One study included patients aged between 9 and 17 years [33,38]. In a single intervention-designed longitudinal study by Vicente et al., 108 participants aged 4–15 years with a main curve more significant than 25 degrees and a Risser 0–3 were examined. They reported that ISJ-3D night braces slowed the increase in angle values and progression of the curve. Female patients were followed up until Risser 4, and male patients were followed up until Risser 5. The mean follow-up period was reported as 2.78 years for males (SD ± 1.85) and 1.97 years for females (SD ± 1.19). An important problem here is that the mean age at the end of treatment was reported as 13.7 ± 1.1 years for males and 13.7 ± 1.2 years for females [13]. If treatment was completed at Risser 5 in males, the mean age should be expected to be older, such as 16 or 17 years [42], while another issue is that a patient who started treatment at the age of 4 years would be expected to have a mean follow-up time of about 10 years when followed up to Risser 4 or 5 values. Such situations seem contradictory in terms of interpreting the study results. 

According to SRS standardization criteria, Risser values of patients included in bracing studies are recommended to be 0–2, which is a child’s fastest growing period [41]. In the nine studies included in this systematic review, a Risser value of 0–2 was determined as the inclusion criterion. A Risser value between 0 and 3 was the inclusion criterion in 4 studies. The Risser value of 0–4 was approved as an inclusion criterion by Heegard et al. [34,36,37] in three retrospective studies and the end results study of a randomized controlled trial [38]. The risk of scoliosis progression in a child with a Risser value of 0 and a high growth potential cannot be the same as the risk of scoliosis progression in a child with a Risser value of 4 who has completed most of his/her maturation [7]. The fact that this difference will impact the study’s findings should be taken into account. In the retrospective cohort study by Janicki et al., the authors reported that the Providence brace was more effective in preventing surgery and curve progression when initial curves were 35 degrees or less, according to the new SRS criteria. However, the authors’ inclusion criteria and patient ages were not compatible. While the authors reported a Risser sign 0, 1, or 2 as inclusion criteria, it was reported that patients aged between 10.3 and 17.2 years were included in the TLSO group and patients aged between 10.5 and 14.9 years were included in the Providence group range [26].

In studies evaluating the effectiveness of brace treatment, follow-up for at least 2 years after maturation is completed is recommended [41]. Among the studies that could be included in this review, only the study by Lee et al. reported that patients were followed up for 2 years after maturation was completed. In the other studies, the mean follow-up period was short and ranged from 6 to 42 months. 

Notably, relatively few pertinent studies were found during the analysis period (1997–2024), and the methodological quality was severely lacking. For example, Price et al. mention that using a night brace should be encouraged; however, the study was a prospective study only, and its methodological strength is weak to offer conclusions. The authors reported that 115 (83%) of the patients who participated in the study showed improvement. However, only 44 patients completed the treatment program. The 139 patients evaluated for inclusion in the study were reported to be between 10 and 16.6 years old, but the mean age of the patients included in the study was not presented. The mean follow-up period of the patients was reported to be one year and seven months. It was reported that brace treatment was followed up for 6–8 months after Risser 4 in female patients and 12–18 months after Risser 4 in male patients. Considering the follow-up period, it can be considered that the patients included in the study were older and the risk of progression was quite low [20]. 

In addition to the fact that the majority of the included studies were retrospective, one of the important methodological problems in the studies is that treatment compliance was not evaluated in most studies. Karol et al. reported unsuccessful results with low patient compliance (38%) in their study examining the efficacy of different types of braces in male patients [22]. In a retrospective study by Gepstein et al., patients with adolescent idiopathic scoliosis treated with the Charleston brace (85 patients) or TLSO brace (37 patients) were analyzed. The compliance was reported to be around 80%. However, although it was stated that the curvatures were measured at the beginning and end of the treatment, the degrees of curvature expected to be expressed as Cobb angle were not presented to give an idea in terms of any comparison or homogeneity of the groups, and the study methodology is weak in these respects and in terms of being retrospective [24]. Ten of the seventeen patients did not comply with their treatment, according to Bohl et al.’s report, and there was a correlation between the rate of surgery and treatment compliance [28]. Furthermore, Capek et al. reported that both the compliance and success of brace use were quite low in their study [35].

Another important point that draws attention in this review study is that in some studies, the efficacy of night braces was evaluated with supine X-rays, while the efficacy of the brace applied to the control group was evaluated with standing X-rays [22,25,31]. This creates a problem for comparing the efficacy of two different braces because it has been reported in many studies that the angle of curvature is lower in supine imaging methods compared to standing imaging methods [43,44,45,46].

Considering the results of these studies, it is interesting to see that several systematic reviews have been conducted on night braces in the treatment of scoliosis, and some conclusions have been drawn from the literature [14,15]. Furthermore, statements that night brace use alone is effective in the treatment of idiopathic scoliosis were also presented, but it was emphasized that further studies including more sociodemographic data are needed [13]. A review of the literature on the subject shows that it is impossible to reach definitive conclusions on the effectiveness of the use of night braces alone in treatment due to methodological deficits, design problems, and other problems related to evaluations based on existing studies [47].

Costa et al. [47] highlight the insufficiency of randomized controlled trials to draw conclusive results on the effects of night-time braces. The study emphasizes the methodological limitations of existing research, which is predominantly retrospective, cautioning against recommending the use of night-time braces based on current evidence. Additionally, the study underscores the importance of considering the positioning of measurements when assessing the efficacy of wearing a night-time brace, indicating a gap in reflecting the true impact of such braces in a horizontal position. However, it is seen that Costa et al. did not examine the contents of the studies they included in their meta-analysis in detail. In their meta-analysis, they included the study of Simony et al. [12], which we did not include in our review study, and reported the success of rigid night braces as 52–89% [47]. Regarding this, a selective study with favorable inclusion criteria was conducted by Simony et al. The paper contains no information regarding the results of patients whose correction effect was less than 60 percent. Remarkably, neither the abstract nor the discussion go into further detail about this specific patient selection. Furthermore, the group of Simony et al. could not satisfy the SRS inclusion requirements for research involving braces [12].

The systematic review by Ruffili et al. published in 2021 evaluating the effect of night bracing in adolescent idiopathic scoliosis included seven studies [16]. The authors did not include some of the studies included in this study that were published before 2021 in their review [20,23,27,28,29,30,31]. A total of 20 studies published up to 2024 evaluating the efficacy of night bracing in patients with adolescent idiopathic scoliosis were included in this systematic review. Comparable to our findings, Ruffili et al. stated that there is not enough information in the literature at this time to allow us to make judgments regarding braces for use at night in the management of adolescent idiopathic scoliosis [16].

Generally, asymmetric techniques with strong corrective effects are applied in the treatment of scoliosis to reverse asymmetric loading on the vertebrae in both brace and exercise approaches, as well as in the control of activities of daily living [1,40,48,49,50]. Night braces are worn only during sleep and do not support the spine during daytime hours. This limited use can result in the spine not constantly receiving the necessary corrective forces. However, it is important to reverse the asymmetrical forces acting on the spine against gravity in daily living activities. A detailed review of the available studies in the literature shows that the results of these studies suggest that night braces may not provide the continuity needed to stop or correct the progression of AIS at risk for being progressive.

The strength of this systematic review study is its detailed examination of the articles’ contents. This study has several limitations The review was limited to papers published in the English, Turkish and Spanish languages, so it is possible that other potentially relevant studies were omitted. The majority of the studies reviewed were retrospectively designed and only one was a randomized controlled trial. A meta-analysis study was not possible due to the lack of randomized controlled trials. Treatment outcomes may also be impacted by variations in the night brace types utilized in the Providence, Charleston, and Boston brace studies. Finally, it is challenging to reach a firm conclusion regarding the long-term effects of night bracing due to the lack of data on long-term outcomes.

## 5. Conclusions

According to the results of this systematic review, the number of randomized controlled trials is insufficient to draw conclusions about the effects of the use of night braces. Furthermore, the studies are controversial in terms of methodological quality, so it is inappropriate to recommend the use of night bracing in management of AIS based on the published research, which is almost all retrospective, and the assessment of the effectiveness of bracing in the horizontal position prevents comparability and a proper interpretation of the results. Additionally, it is seen that the follow-up periods of studies with night braces are too short to decide on the success rate. In order to optimize treatment outcomes in AIS, it is very important to take into account various factors such as patient compliance, additional exercises and the type of brace used, and the results could not be isolated in terms of the effects of these conditions. 

Well-designed prospective RCT studies with comparable unselected samples are needed to clarify this subject’s information and reach a definitive conclusion. In order to standardize studies, parameters such as age, Risser sign, Cobb angle, gender, treatment compliance and even progression factor estimation should be determined and optimal methods should be determined through studies with long-term follow-up and comparison of different treatment applications.

## Figures and Tables

**Table 1:** Characteristics of the studies included in this review.

	Year/Country	Type of Brace/Intervention	Study Time/Sample Size	Gender	Skeletal Maturity/Age	Cobb Angle°	Outcome Measurements	Brace wearing Time	Follow-Up	Survival Rates
Katz et al. [19]	1997, Texas	Boston Charleston	Retrospective *n* = 153 *n* = 166	243 Females 25 Males	Risser 0–2, ≥10 years	25°–45°	Cobb Surgery rate Curve progression	Not specified	34 mos	41% for Charleston 61% for Boston
Price et al. [20]	1997, USA	Charleston	Prospective *n* = 98	90 Females 8 Males	Risser 0–2, ≥10 years	25°–49°	Cobb Curve Progression	Night-time	3.5 years	
Howard et al. [21]	1998, Canada	TLSO Charleston Milwaukee	Retrospective *n* = 45 *n* = 95 *n* = 35	NA	≥10 years		Cobb Surgery rate Curve Progression	Fulltime Night-time Fulltime	20 mos 16 mos 19 mos	55% for TLSO 48% for Charleston 35% for Milwaukee
Karol et al. [22]	2001, Texas	Milwaukee Charleston Boston	Retrospective and prospective *n* = 10 *n* = 53 *n* = 54	117 Males	Risser 0–3, ≥ 10 years	18°–45°	Cobb Curve Progression Surgery rate Compliance with Treatment	-	3.1 years	26% for all braces
D’Amato et al. [23]	2001, Rhode Island	Providence	Prospective *n* = 102	102 Females	Risser 0-2, >10 years	20°–42°	Cobb Curve Progression	8 h/night	>2 years	74%
Gepstein et al. [24]	2002, Israel	Charleston TLSO	Retrospective *n* = 87 *n* = 37	94 Females 28 Males	10–16 years	-	Cobb Curve Progression or Surgery rate	8 h/day 18–22 h/day	24 mos	81.2% for Charleston 82.1% for TLSO
Yrjonen et al. [25]	2006, Finland	Providence Boston	Prospective *n* = 36 *n* = 36	72 Females	Risser 0–3, 10–15 years	>25°	Cobb Curve progression	Night-time 23 h/day	1.8 years	68.6%
Janicki et al. [26]	2007, Canada	TLSO Providence	Retrospective *n* = 48 *n* = 35	83 Females	Risser 0–2, >10 years	25°–40°	Cobb Curve Progression Surgery rate	22 h/day 8–10 h/night	2 years	15% for TLSO 31% for Providence
Lee et al. [27]	2012, Korea	Charleston	Retrospective *n* =95	87 Females 8 Males	Risser 0–2, >10 years	25°–40°	Cobb Curve Progression Surgery rate	8 h/night	>2 years	77.9%
Bohl et al. [28]	2014, ABD	Providence	Retrospective *n* = 34	29 Females 5 Males	Risser 0–2, >10 years	25°–40°	Cobb Surgery rate Curve Progression	8 h/day	-	29%
Ohrt-Nissen et al. [29]	2016, Denmark	Providence	Retrospective *n* = 63	75 Females 2 Males	Risser 0–2, >10 years	25°–40°	Cobb Curve progression HRQOL	Night-time	2 years	57%
Thompson et al. [30]	2017, USA	TLSO Boston	Retrospective *n* = 168	NA	Risser 0–2, >10 years	25°–45°	Cobb Curve Progression Surgery rate	12.2–13.3 h/day	22 mos	64.2–84.6%
Davis et al. [31]	2018, USA	Providence	Retrospective *n* = 56	51 Females 5 Males	Risser 0–2, 10–18 years	25°–40°	Cobb Curve Progression Surgery rate	Night-time	2.21 years	57.1%
Ohrt-Nissen et al. [32]	2019, Denmark	Boston Providence	Retrospective *n* = 37 *n* = 40	75 Females 2 Males	Risser 0–2, >10 years	25°–40°	Cobb	>18 h/day 8 h/day	25 mos	65% for Boston 57% for Providence
Vicente et al. [13]	2021, Spain	Providence	Longitudinal study *n* = 108	94 Females 14 Males	Risser 0–3, 4–15 years	>25°	Cobb Surgery Rate	8–10 h/night	1 year	NA
Dufvenberg et al. [33]	2021, Sweden	Boston Specific exercises Physical activity	RCT *n* = 45 *n* = 45 *n* = 45	111 Females 24 Males	9–17 yr	25°–40°	Cobb ATR HRQoL, perception of spinal appearance Physical activity	Night-time	6 mos	NA
Heegaard et al. [34]	2023, Denmark	Providence	Retrospective *n* = 135	116 Females 19 Males	Risser 0–4, >10 years	25°–40°	Cobb Curve Progression Surgery rate	Night-time	13-39 mos	39% for Risser 0–2 63% for Risser3–4
Capek et al. [35]	2023, Sweden	Providence Boston	Retrospective *n* = 249 *n* = 109	314 Females 44 Males	Risser 0–3, >10 years	20°–40 °	Cobb Curve Progression Surgery rate Compliance with Treatment	Nightime 23 h	1.45 years	45% for Providence 54% for Boston
Heegaard et al. [36]	2024, Denmark	Providence	Retrospective *n* = 146	127 Females 19 Males	Risser 0–4	25°–45°	Cobb Curve Progression Surgery rate Sagital Plane	Night-time	1year after treatment	17%
Heegaard et al. [37]	2024, Denmark	Providence	Retrospective *n* = 299	271 Females 28 Males	Risser 0–4	25°–55°	Cobb Curve Progression Surgery rate	8 h/night	-	25% for >40° 62% for 40°>
Charalampidis et al. [38] (End results of the study by Dufvenberg et al.)	2024, Sweden	Boston Specific exercises Physical activity	RCT *n* =42 *n* =39 *n* = 41	111 Females 24 Males	Risser 0–4, 9–17 years	25°–40°	Curve Progression Surgery rate	8 h/night	12.9 mos 16.2 mos 16.1 mos	76% for Boston 53% for Exercise 58% for Physical activity

RCT: randomized controlled trial, h: hour, NA: not available.

## Data Availability

The data presented in this study are available on request from the corresponding author.
